# Identification and validation of necroptosis-related gene signatures to predict clinical outcomes and therapeutic responses in acute myeloid leukemia

**DOI:** 10.18632/aging.205231

**Published:** 2023-11-21

**Authors:** Xiang-Mei Wen, Zi-Jun Xu, Ji-Chun Ma, Pei-Hui Xia, Ye Jin, Xin-Yi Chen, Wei Qian, Jiang Lin, Jun Qian

**Affiliations:** 1 Laboratory Center, Affiliated People’s Hospital of Jiangsu University, Zhenjiang 212002, Jiangsu, P.R. China; 2 Zhenjiang Clinical Research Center of Hematology, Affiliated People’s Hospital of Jiangsu University, Zhenjiang 212002, Jiangsu, P.R. China; 3 The Key Lab of Precision Diagnosis and Treatment in Hematologic Malignancies of Zhenjiang City, Affiliated People’s Hospital of Jiangsu University, Zhenjiang 212002, Jiangsu, P.R. China; 4 Department of Hematology, Affiliated People’s Hospital of Jiangsu University, Zhenjiang 212002, Jiangsu, P.R. China; 5 Department of Otolaryngology-Head and Neck Surgery, Affiliated People’s Hospital of Jiangsu University, Zhenjiang 212002, Jiangsu, P.R. China

**Keywords:** necroptosis, acute myeloid leukemia, prognosis, tumor microenvironment, immune infiltration, immunotherapy

## Abstract

Background: Necroptosis is a tightly regulated form of necrotic cell death that promotes inflammation and contributes to disease development. However, the potential roles of necroptosis-related genes (NRGs) in acute myeloid leukemia (AML) have not been elucidated fully.

Methods: We conducted a study to identify a robust biomarker signature for predicting the prognosis and immunotherapy efficacy based on NRGs in AML. We analyzed the genetic and transcriptional alterations of NRGs in 151 patients with AML. Then, we identified three necroptosis clusters. Moreover, a necroptosis score was constructed and assessed based on the differentially expressed genes (DEGs) between the three necroptosis clusters.

Results: Three necroptosis clusters were correlated with clinical characteristics, prognosis, the tumor microenvironment, and infiltration of immune cells. A high necroptosis score was positively associated with a poor prognosis, immune-cell infiltration, expression of programmed cell death 1/programmed cell death ligand 1 (PD-1/PD-L1), immune score, stromal score, interferon-gamma (IFNG), merck18, T-cell dysfunction-score signatures, and cluster of differentiation-86, but negatively correlated with tumor immune dysfunction and exclusion score, myeloid-derived suppressor cells, and M2-type tumor-associated macrophages. Our observations indicated that a high necroptosis score might contribute to immune evasion. More interestingly, AML patients with a high necroptosis score may benefit from treatment based on immune checkpoint blockade.

Conclusions: Consequently, our findings may contribute to deeper understanding of NRGs in AML, and facilitate assessment of the prognosis and treatment strategies.

## INTRODUCTION

Acute myeloid leukemia (AML) is a highly lethal hematological malignancy. It is characterized by proliferative enhancement, blocked differentiation, and dysregulated apoptosis [1]. Intensive induction chemotherapy is first-line treatment for AML [2]. Conventional types of chemotherapy can induce remission in some patients, but most patients experience a relapse of AML [3]. For decades, many novel targeted therapies have been developed, but the prognosis for AML remains poor, with 5-year survival of ~10% [4, 5]. Also, childhood AML has an unfavorable prognosis, and the prevalence of relapse is high [6]. Therefore, identifying new molecular profiles that can predict the prognosis and aid development of new therapeutic targets against AML is important.

Necroptosis is a type of regulated cell death characterized by loss of plasma-membrane integrity and escape of cellular contents that is independent of caspases, the morphological characteristics of necrosis, and instigation of an inflammatory response [7–10]. The main mediators of necroptosis execution are receptor interacting protein kinase 1 (RIPK1) and RIPK3, and mixed lineage kinase domain-like protein [8, 11]. Recently, several studies have demonstrated the influence of necroptosis on tumorigenesis, progression, and metastasis in various types of cancer [12, 13]. The pro-tumorigenic or antitumorigenic effects of RIPK3-mediated necroptosis are dependent upon the type of cancer and conditions during tumorigenesis. It has been reported that RIPK3 deficiency does not alter MYC-driven lymphomagenesis or the killing of malignant lymphoma cells induced by chemotherapeutics [14]. Moreover, RIPK3 expression is downregulated and correlates with poor clinical outcomes in AML [12, 15]. However, the key mediators of the necroptotic pathway (alone or in combination) have been shown to enhance neoplastic progression and metastasis [16, 17].

Necroptosis is a type of inflammatory cell death that contributes to innate immunity and shapes subsequent adaptive immunity [18, 19]. The machinery of necroptotic cell death promotes immune responses by increasing secretion of cytokines and chemokines [20, 21]. RIPK1 signaling and activation of nuclear factor-kappa B may be necessary during necroptotic cell death to result in efficient cross-priming and antitumor immunity [22]. Nevertheless, necroptotic tumor cells also attract dendritic cells and macrophages that can further enhance immunosuppression [23]. Thus, necroptosis can shape adaptive immunity against tumor progression and generate an immunosuppressive tumor microenvironment (TME).

Due to technical limitations, most studies have investigated only one or two necroptosis-related genes (NRGs). Numerous genes interact with each other and with environmental factors in a highly coordinated manner. The signatures of novel NRGs for the prognosis or TME of hepatocellular carcinoma, colon cancer, bladder cancer, pancreatic cancer, and cutaneous melanoma have been identified [24–28]. However, the prognostic role of the NRG signature in AML has not been elucidated. We explored the association of multiple NRGs with the prognosis of AML and cell infiltration into the TME. We aimed to provide insights into tumorigenesis and open-up a novel therapeutic strategy for AML.

## MATERIALS AND METHODS

### Data acquisition

The raw data of transcriptome profiling and corresponding clinical information of 151 AML samples were downloaded from the Genomic Data Commons of The Cancer Genome Atlas (TCGA) portal (https://portal.gdc.cancer.gov/repository). Since normal samples were not included in AML from TCGA database, we collected 70 bone marrow (BM) normal samples from the GTEx database (http://www.GTExportal.org/home/). Batch effects between two datasets were corrected using the “ComBat” method from the sva package. Detailed information on these AML patients is shown in Supplementary Table 1. Data on the somatic gene mutations and gene copy number variations (CNVs) of AML patients were also obtained from TCGA database. Data on somatic mutations were analyzed with the “mafCompare” function in the “Maftools” package [29]. Significant amplifications or deletions of the copy number were detected by filtered segmented copy number data (Affymetrix SNP 6.0 platform) using the GISTIC2.0 algorithm [30]. We also collected six AML datasets (GSE6891, GSE10358, GSE12417_UA, GSE12417_UP, GSE37642_UA, and GSE37642_UP), combining them using the “ComBat” algorithm, in order to validate the consensus clustering results in TCGA.

### Consensus molecular clustering for 67 NRGs

A list of NRGs was collected from the work of Zirui Zhao and colleagues [31]. All 67 genes are provided in Supplementary Table 2. We applied the “ConsensusClusterPlus” package for consensus clustering and distinguishing patients into three distinct necroptosis clusters based on these 67 NRGs [32]. Consensus clustering is an established unsupervised classification method for data analyses. The appropriate cluster number (k) was calculated from the relative change in area under the cumulative distribution function. Plots were created to ascertain if they are consistent or inconsistent for various values of k.

### Identification of differentially expressed genes (DEGs)

DEGs were identified among three necroptosis subtypes via the “limma” package in R 4.0.4 (R Institute for Statistical Computing, Vienna, Austria; http://www.r-project.org/). Specifically, with the “limma-voom” package, we normalized gene expression, which was then fed into “lmFit” and “eBayes” functions in the limma package. A total of 829 DEGs were identified. Patients were divided into three gene clusters via unsupervised clustering of DEGs.

### Generation of a necroptosis score

We also used principal component analysis (PCA) to evaluate the necroptosis pattern for each individual. First, univariate Cox regression analysis was undertaken on 829 DEGs to identify prognosis-related genes. Second, we conducted recursive feature elimination with 10-fold cross-validation in the 361 genes that had a significant prognostic impact. Third, we carried out PCA to construct a signature of the relevant genes of necroptosis with principal component (PC)1 and 2 as signature scores. Accordingly, we calculated a necroptosis score [33]:


Necroptosis score=∑(PC1i+PC2i)

where *i* is the expression of NRGs.

AML patients were divided into a low-score group and high-score group according to the maximally selected rank statistics (maxstat) method.

### Clinical features and analyses of signaling-pathway enrichment

We examined the relationships between different AML subtypes and clinicopathological characteristics, including French–American–British (FAB) subtypes, cytogenetic risk, sex, age, and white blood cell count. To explore biological information and protein functions, enrichment analyses were done with the “clusterprofile” package in R using the Gene Ontology (GO) database (http://www.geneontology.org/). We detected significant differences in signaling pathways between different groups using the R package “GSVA”.

### Estimation of infiltration of TME cells and prediction of immune responses

We adopted the CIBERSORT algorithm to quantify infiltration of immune cells in different NRGs patterns [34]. As part of this analysis, the ESTIMATE algorithm was used to analyze the tumor purity, immune score, and stromal score [35]. We also analyzed the correlations between expression of programmed cell death 1/programmed cell death ligand 1 (PD-1/PD-L1) and the necroptosis score. We collected four independent immunotherapy cohorts including four cancer types to determine difference in immune checkpoint blockade (ICB) responsiveness between patients with high and low necroptosis score. The response information was downloaded from the supplementary data of the respective papers.

### Statistical analyses

R was employed for statistical analyses. Kaplan–Meier survival curves were used to assess survival differences between patient groups using the “survminer” package. Categorical data were compared using the chi-square test or Fisher’s exact test. The correlation between continuous variables was compared using Spearman’s rank correlation test. Data visualization was undertaken using the R packages “ggplot2”, “circlize” (for Circos plots), and Maftools (for co-onco plots or forest plots). *P* < 0.05 was considered significant.

### Data and code availability

The code files (using R) employed to reproduce the figures contained within the manuscript are available upon reasonable request. The original contributions presented in the study are provided in the Article/Supplementary Materials.

## RESULTS

### “Landscape” of genetic and transcriptional alterations of NRGs in AML

**Figure 1:**
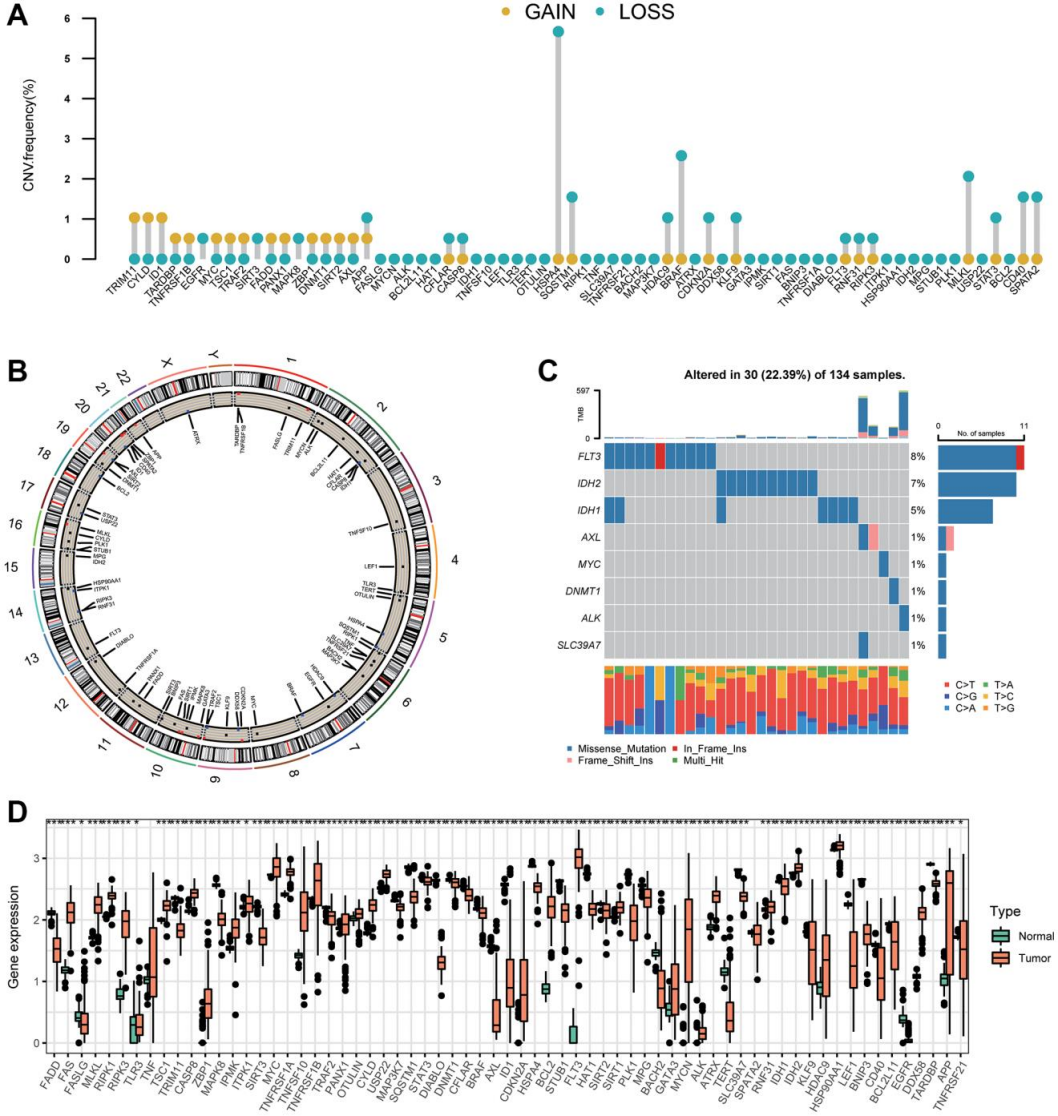
**Variation in expression of necroptosis-related genes in AML.** (**A**) The CNV frequencies of 67 necroptosis-related genes. The frequencies of amplification and deletion are labeled as orange dots and green dots, respectively. (**B**) The position of the CNV alteration of necroptosis-related genes on 23 chromosomes. (**C**) Mutation frequency of the top-eight necroptosis-related genes in 134 patients with AML. (**D**) A boxplot indicating expression of the 67 necroptosis-related genes between normal samples and AML samples (^*^P < 0.05, ^**^P < 0.01, ^***^P < 0.001).

We assessed 67 NRGs (Supplementary Table 1). To reveal chromosomal gains and losses, we applied a somatic CNV analysis. The CNV of NRGs was not prevalent in AML. TRIM11, CYLD, and ID1 were involved primarily in gene amplification, whereas deletion of HSPA4, BRAF, and SQSTM1 was common (Figure 1A, Supplementary Table 2). The location of CNV alteration of 67 NRGs on chromosomes is illustrated in Figure 1B (Supplementary Tables 3, 4). Normal samples and tumor samples could be distinguished clearly into two distinct groups via PCA (Supplementary Figure 1A). Also, the prevalence of somatic mutations of NRGs in AML was not widespread, and the top-eight genes with altered expression were FLT3 (8%), IDH2 (7%), IDH1 (5%), AXL (1%), MYC (1%), DNMT1 (1%), ALK (1%), and SLC39A7 (1%) (Figure 1C). We also compared mRNA expression between AML samples and normal samples. All 67 NRGs had remarkable differences in mRNA expression between AML samples and normal samples except for TNF and SPATA2 (Figure 1D). Besides, NRGs exhibited distinct patterns in different immune cell types, myeloid malignancies, and lymphoid malignancies (Supplementary Figure 1B, 1C). Relatively higher expression of FLT3 was detected compared with that of other NRGs in AML samples (Supplementary Figure 1C).

### Identification of three necroptosis clusters in AML

**Figure 2:**
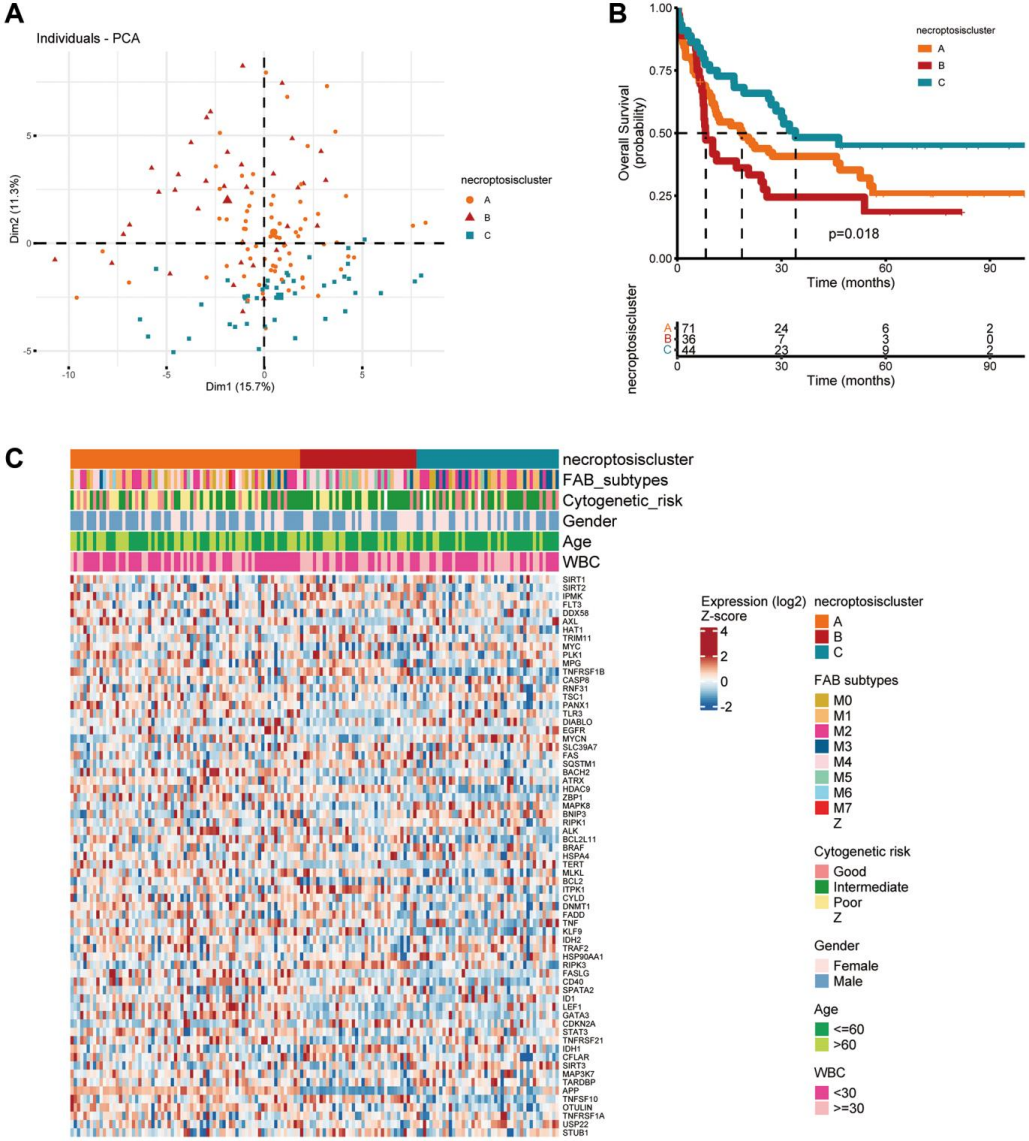
**Subtypes of necroptosis-related genes and their clinicopathological and biological characteristics in AML.** (**A**) PCA of transcriptomes among the three identified clusters. (**B**) Kaplan–Meier curves showing overall survival for the three necroptosis clusters. (**C**) Clinicopathological characteristics and expression of necroptosis-related genes among the three necroptosis clusters.

Survival information of 151 patients from TCGA dataset was used for analyses (Supplementary Table 5). Univariate Cox regression analyses were used to investigate the prognostic value of NRGs (Supplementary Table 6). A consensus clustering analysis based on expression of NRGs was conducted for AML. The optimal k value was 3 (Supplementary Figure 2A–2C). According to PCA, three clusters had significant differences in their gene-expression profiles (Figure 2A). The entire cohort was clustered into necroptosis cluster A (*n* = 71), necroptosis cluster B (*n* = 36), and necroptosis cluster C (*n* = 44) (Figure 2B). Kaplan–Meier survival curves for overall survival (OS) indicated significant differences among the three clusters. Patients with necroptosis cluster B had a poor survival outcome (*P* = 0.018) (Figure 2B). Moreover, the three clusters were confirmed by performing consensus clustering analysis in combined AML cohorts encompassing six datasets (1115 patients), although the prognostic impact was not validated (Supplementary Figure 3). Then, the clinicopathological characteristics of the three necroptosis subtypes were compared. Cases with FAB M3 was mainly observed in patients with necroptosis cluster C. Necroptosis cluster B was not associated with good cytogenetic risk and APP was barely expressed in necroptosis cluster B. In terms of gender, age and WBC, no differences were observed among three clusters (Figure 2C).

### Associations between the TME and three necroptosis clusters

**Figure 3:**
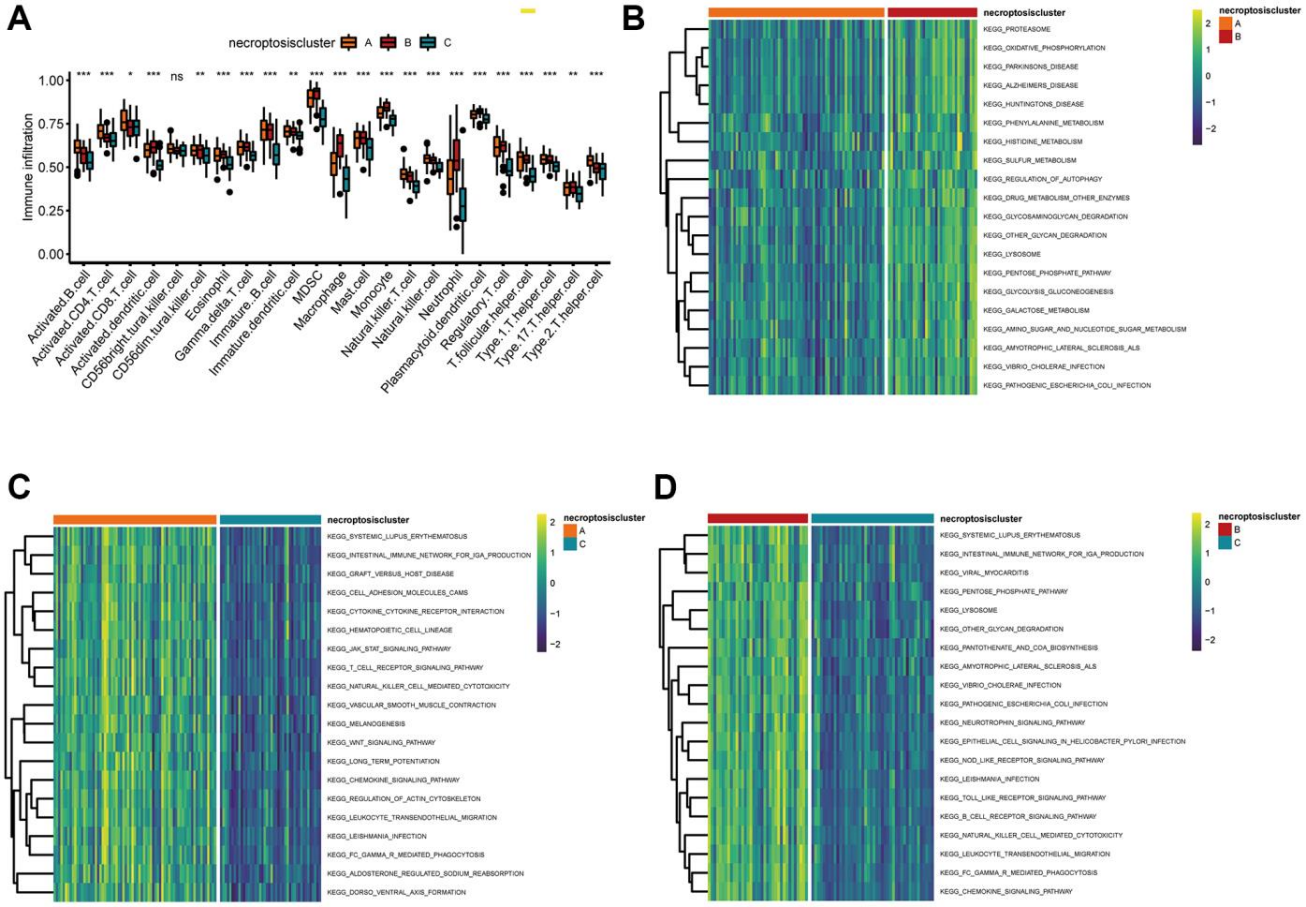
**Correlations between the TME and three necroptosis clusters.** (**A**) Analyses of tumor-infiltrating immune cells in the three necroptosis clusters. ^*^P < 0.05, ^**^P < 0.01, ^***^P < 0.001, ns, not significant. (**B**–**D**) Heatmap of the enrichment analyses in three necroptosis clusters using the GSVA package. (**B**) Cluster A vs. cluster B; (**C**) cluster A vs. cluster C; (**D**) cluster B vs. cluster C.

Using the CIBERSORT algorithm, we explored the profiles of 23 types of infiltrating immune cells in three necroptosis clusters. Significant differences in immune-cell infiltration were noted except for cluster of differentiation (CD)56 bright natural killer cells among the three types of necroptosis clusters (Figure 3A). Necroptosis cluster B had the highest number of infiltrating macrophages. Enrichment analyses using the GSVA package were also done to elucidate the biological characteristics among the three necroptosis clusters. Necroptosis cluster B was strongly related to metabolic pathways, including “phenylalanine metabolism”, “histidine metabolism”, and “sulfur metabolism” (Figure 3B, Supplementary Table 7). Necroptosis cluster A showed significant enrichment in immune system-related pathways such as “systemic lupus erythematosus”, “intestinal immune network for IgA production”, “cell adhesion molecules”, “cytokine receptor interaction”, “T cell receptor signaling pathway”, “natural killer cell-mediated cytotoxicity”, and “chemokine signaling pathway activation” (Figure 3C, Supplementary Table 8). Necroptosis cluster B was also highly associated with immune system-related pathways, including “intestinal immune network for IgA production”, “NOD-like, and Toll-like receptor signaling pathway”, “B cell receptor signaling pathway”, and “natural killer cell-mediated cytotoxicity” (Figure 3D, Supplementary Table 9).

### Identification of three gene clusters in AML

**Figure 4:**
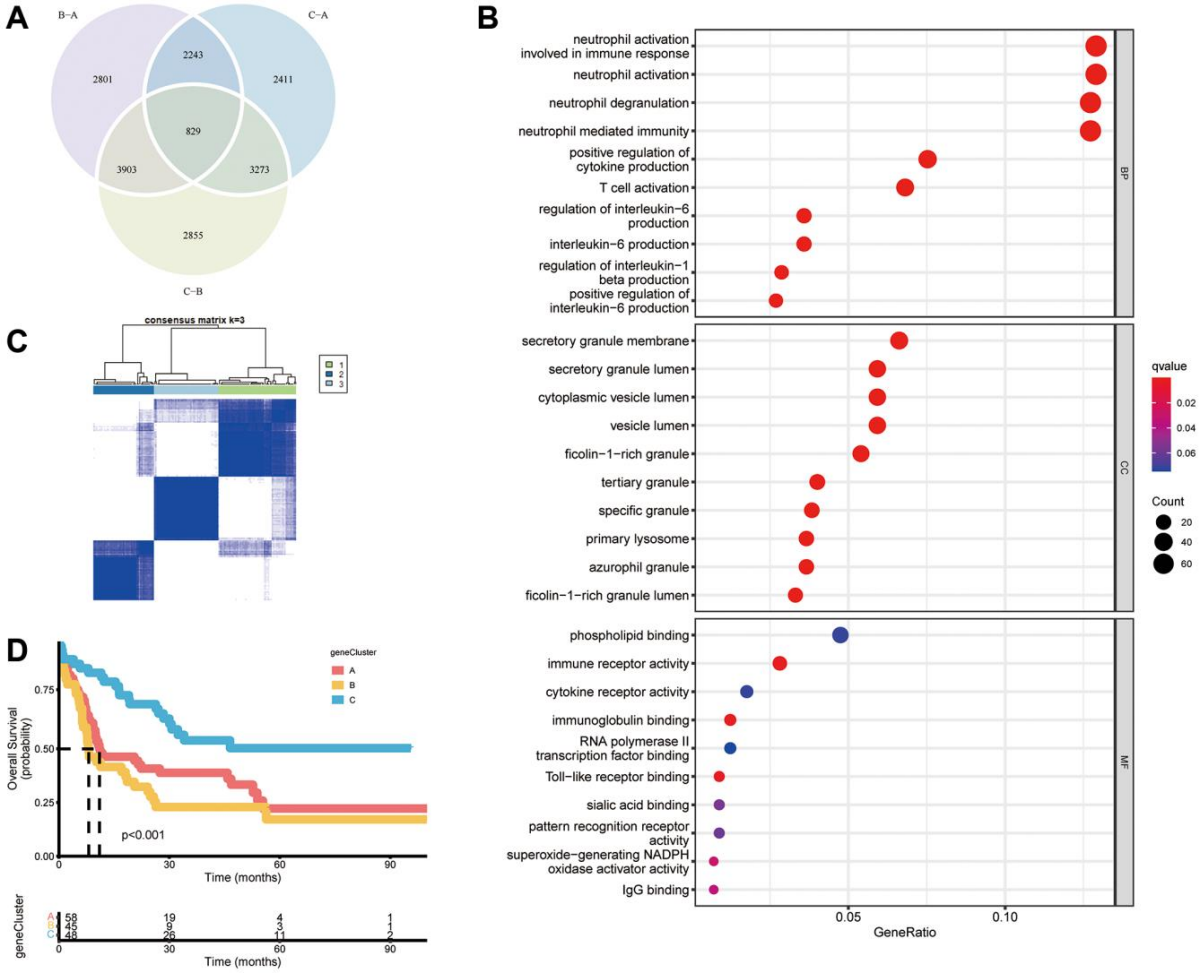
**Construction of gene subtypes based on DEGs.** (**A**) Venn diagram of 829 necroptosis-related DEGs among three necroptosis clusters. (**B**) Analyses of functional enrichment of DEGs using the GO database. (**C**) Three gene clusters were categorized by a consensus matrix heatmap (k = 3). (**D**) Kaplan–Meier curves of overall survival for three gene clusters (*P* < 0.001, log-rank test).

**Figure 5:**
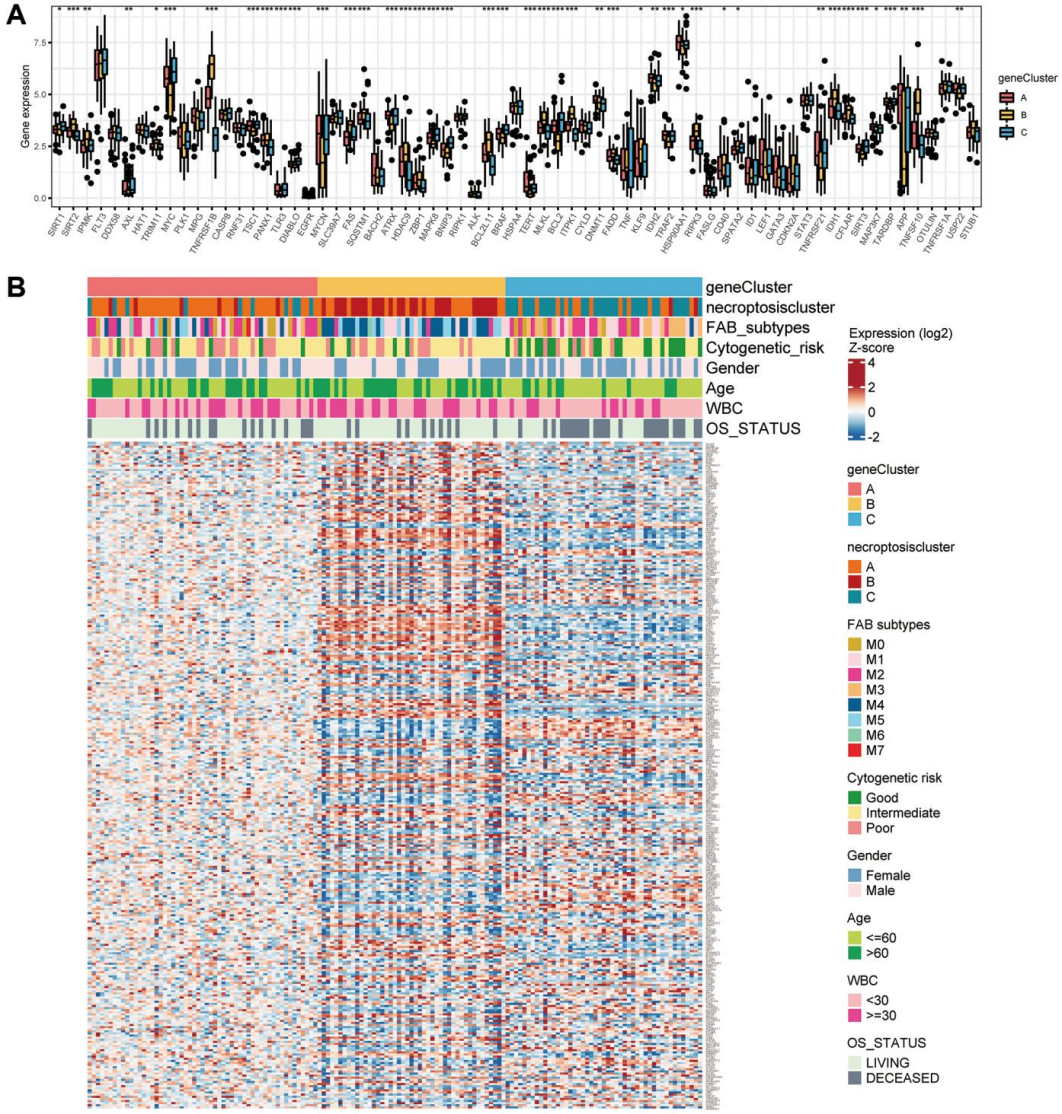
**Correlations between the TME and three gene clusters.** (**A**) Gene expression of 67 necroptosis-related genes among the three gene clusters. (**B**) Association of clinicopathologic features with the three gene clusters.

We wished to further investigate the underlying biological functions of the three clusters. We generated a Venn diagram to illustrate the overlapped DEGs, and a set of 829 genes was screened out (Figure 4A). Subsequently, functional analyses were done using the GO database. Expression of necroptosis subtype-related genes associated with immunity was increased significantly (Figure 4B, Supplementary Table 10), which indicated that necroptosis may participate in regulation of the immune function of the TME. We wished to identify the genes associated with the prognosis among these 829 common DEGs. Hence, we undertook univariate Cox regression analysis and selected 316 genes with *P* < 0.05 to use in subsequent analyses (Supplementary Table 11). Three gene clusters were identified using consensus clustering, and we named them as gene clusters A, B, and C (Figure 4C). Kaplan–Meier curves for OS showed that patients with gene cluster A or gene cluster B had a worse outcome than that for patients with gene cluster C (*P* < 0.001, log-rank test) (Figure 4D). Moreover, the three gene clusters were confirmed using consensus clustering analysis in combined AML cohorts encompassing six datasets (1115 patients), and the prognostic impact was validated (*P* < 0.001, log-rank test) (Supplementary Figure 4A, 4B). Among the three gene clusters, there were significant differences in expression of NRGs such as SIRT1, SIRT2, IPMK, and AXL (Figure 5A). Necroptosis gene cluster C correlated with good cytogenetic risk. More interestingly, these 316 genes showed high expression in gene cluster B (Figure 5B).

### Construction and validation of a necroptosis score

**Figure 6:**
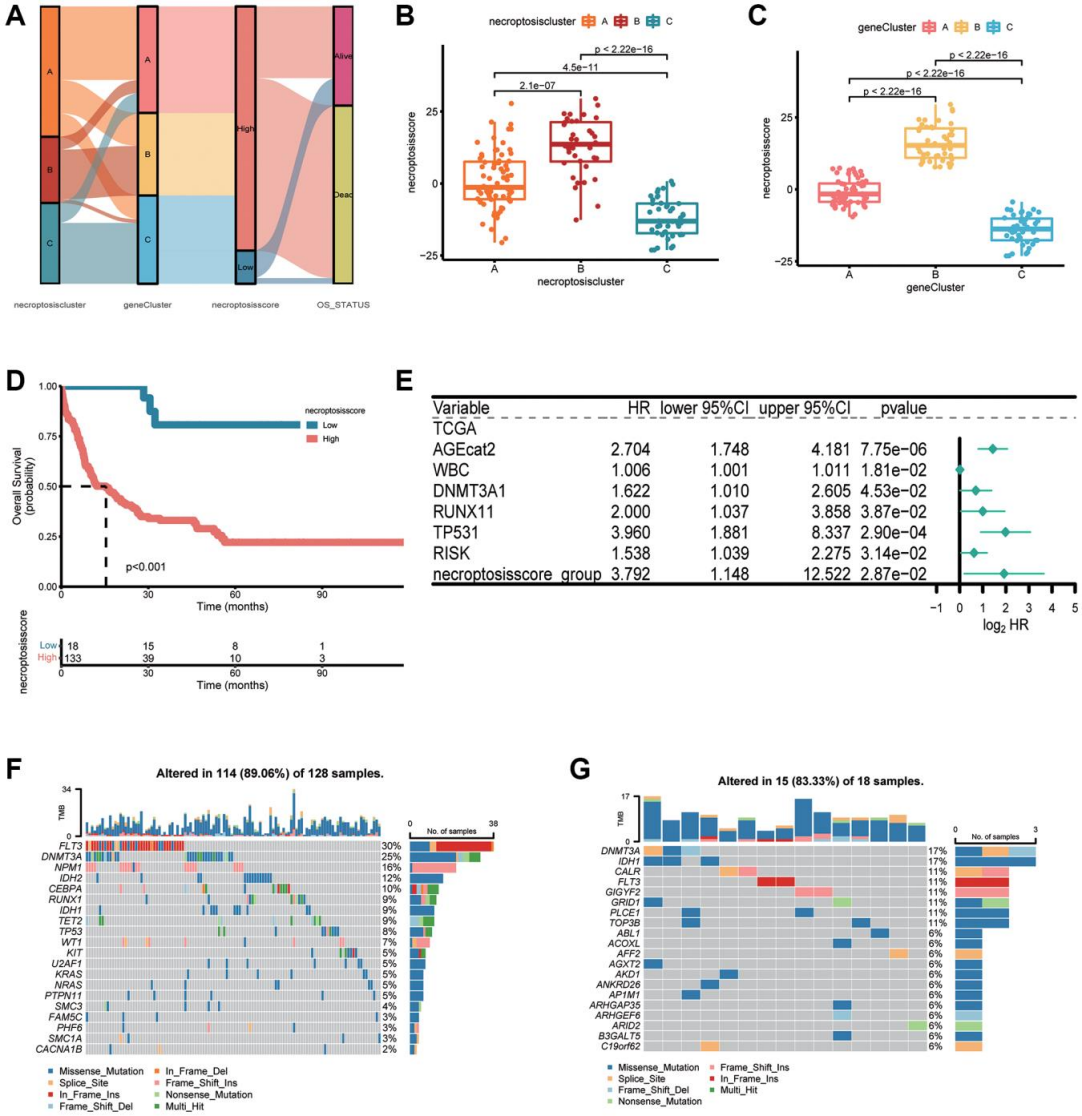
**Construction of a necroptosis score and its clinical relevance.** (**A**) Alluvial plot depicting subtype distributions in groups with different necroptosis clusters, gene clusters, necroptosis score, and overall survival. (**B**) Differences in the necroptosis score between three necroptosis clusters. (**C**) Differences in the necroptosis score between three gene clusters. (**D**) Kaplan–Meier survival analysis of groups with a high necroptosis score or low necroptosis score (*P* < 0.001, log-rank test). (**E**) Multivariate Cox regression analyses of the overall survival of AML patients. (**F**, **G**) “Waterfall” plot of somatic mutation features in groups with the high necroptosis score (**F**) or low necroptosis score (**G**).

We applied a PCA algorithm to calculate the necroptosis score and quantify necroptosis patterns among AML patients because of the complexity and individual heterogeneity in necroptosis modification (Supplementary Table 12). The alluvial plot (Figure 6A) illustrated the distribution of the three necroptosis clusters, three necroptosis gene clusters, necroptosis score, and OS status. Cluster B had a higher necroptosis score than that of cluster A or cluster C (Figure 6B). The necroptosis score of gene cluster B was higher than that of gene cluster A and gene cluster C (Figure 6C). We conducted OS analyses using Kaplan–Meier curves. Patients with a high necroptosis score had a significantly poor prognosis than those with a low necroptosis score (*P* < 0.001, log-rank test) (Figure 6D). Furthermore, the prognostic impact was validated in combined AML cohorts encompassing six datasets (1115 patients) (*P* < 0.001, log-rank test) (Supplementary Figure 4C). Univariate Cox regression analyses revealed a high necroptosis score to be significantly related to shorter OS (HR = 7.673, *P* < 0.01) (Supplementary Table 13). Multivariate Cox regression analyses for OS in TCGA dataset confirmed the necroptosis score to be an independent prognostic biomarker in AML (HR = 3.792, *P* = 0.0287) (Figure 6E). We analyzed the most prevalent somatic mutations for a high score and low necroptosis score to study differences in distribution of somatic mutations between them. FLT3 (30% vs. 11%) and DNMT3A (25% vs. 17%) had a higher prevalence of somatic mutations in the group with a high necroptosis score (Figure 6F, 6G).

### Necroptosis score in immunotherapy

**Figure 7:**
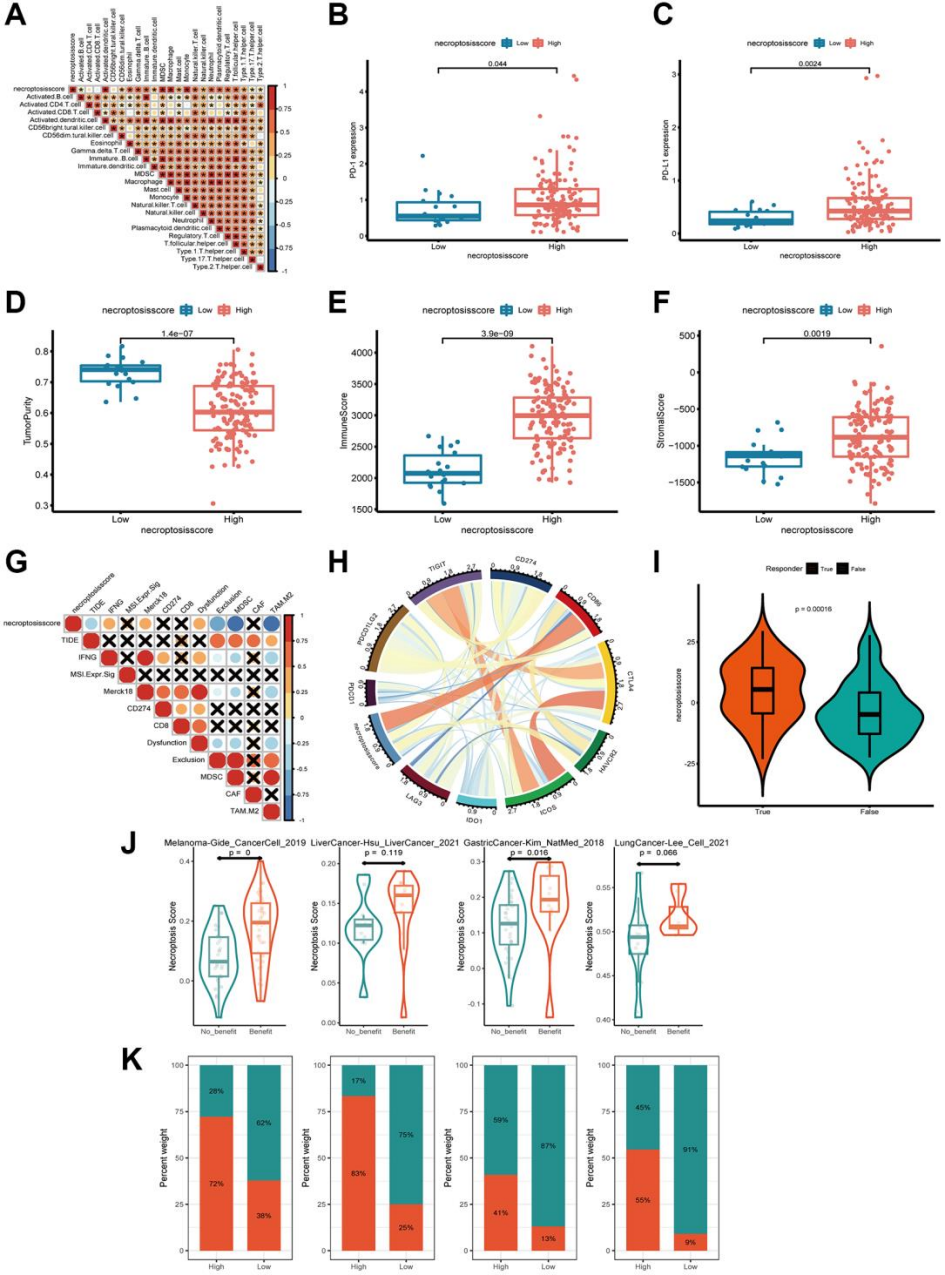
**Exploration of the response of the necroptosis score to immunotherapy.** (**A**) Spearman correlation analysis of tumor-infiltrating lymphocytes and necroptosis score. (**B**, **C**) Expression of PD-L1 and PD-1 in groups with a high necroptosis score (**B**) and low necroptosis score (**C**). (**D**–**F**) Tumor purity, immune score, and stromal score of necroptosis-score groups were analyzed and plotted. (**G**, **H**) Correlations between necroptosis and other immune checkpoints in AML. (**I**) Profile of the necroptosis score in the non-responder group and responder group. (**J**, **K**) Patients with a higher necroptosis score have a higher response to ICB in four independent ICB cohorts compassing of four cancer types.

Next, we investigated if the necroptosis score could be used to predict the response to immunotherapy by patients. We analyzed the correlation between the abundance of immune cells and necroptosis score. The number of activated dendritic cells, gamma delta T cells, macrophages, mast cells, and natural killer T cells was positively related to the necroptosis score (Figure 7A and Supplementary Table 14). The group with a high necroptosis score had upregulated expression of PD-1 and PD-L1, which impaired antitumor immunity further (Figure 7B, 7C).

Based on these findings, we aimed to estimate the overall number of infiltrating immune cells and stromal cells in the groups with a high necroptosis score and low necroptosis score, respectively. We used the ESTIMATE algorithm to calculate tumor purity, immune score, and stromal score. There was a tendency for the group with a high necroptosis score to have lower tumor purity, higher immune score and stromal score (Figure 7D–7F). These data indicated that the group with a high necroptosis score was enclosed by more nontumor components. To identify the group that may be a candidate for immunotherapy, we analyzed the response to immunotherapy based on the necroptosis score. We observed a strong negative correlation between the necroptosis score and T-cell exclusion signatures, including tumor immune dysfunction and exclusion core, myeloid-derived suppressor cells (MDSCs) and the M2 subtype of tumor-associated macrophages (TAMs). The opposite trend was observed among interferon-gamma (IFNG), merck18, and T-cell dysfunction-score signatures (Figure 7G). These findings demonstrated indirectly that the necroptosis score had a critical role in mediating the immune response, and that the group with a high necroptosis score was potentially more sensitive to immunotherapy.

Moreover, we investigated the correlation between the necroptosis score and a collection of genes associated with checkpoints in AML. CD86 was constantly associated with the necroptosis score according to Circos plots (Figure 7H). More excitingly, the necroptosis score was significantly higher in responders than in non-responders, as classified by the TIDE algorithm (http://tide.dfci.harvard.edu/) (Figure 7I). We next investigated whether the necroptosis signatures could predict patients’ response to ICB therapy in real-world immunotherapy cohorts. Using four independent ICB cohorts compassing of four cancer types (Figure 7J), we found that patients with a response to ICB had consistently higher necroptosis score than patients with no response and that the high-necroptosis-score group presented a better response to ICB (Figure 7J, 7K). Hence, patients with a high necroptosis score tended to benefit more from treatment based on immune-checkpoint blockade (ICB).

## DISCUSSION

Necroptosis may prevent or promote the progression of tumor cells [36]. Höckendorf and colleagues identified RIPK3 as a key tumor suppressor in AML [15]. Paradoxically, high expression of RIPK3 leads to productive proliferation and necrotic vulnerability in recurrent breast cancer [37]. Also, necroptosis-induced chemokine ligand 1 (CXCL1) expression may be crucial for the progression of pancreatic ductal adenocarcinoma and promote a macrophage-induced adaptive immune response [38]. Most studies have focused only on the effect of a single NRG or single TME cell type. Hence, we aimed to reveal the clinical characteristics and pattern of infiltration of TME cells mediated by multiple NRGs. Identifying the role of NRGs in the TME could provide important molecular insights into the interactions between necroptosis and the anti-tumor immune response, and facilitate development of more efficacious therapeutic strategies.

Sample classification is a widely applied method based on predefined gene sets. In the present study, 151 AML patients were classified into three subtypes according to expression of NRGs. Necroptosis cluster B carried a worse prognosis and was closely associated with immune system-related pathways, including “intestinal immune network for IgA production”, “NOD-like, and Toll-like receptor signaling pathway”, “B cell receptor signaling pathway”, and “natural killer cell-mediated cytotoxicity”. Simultaneously, the three necroptosis clusters differed significantly in terms of the characteristics of infiltration of immune cells. Based on the DEGs among the three necroptosis clusters, patients were classified into three gene clusters. Furthermore, on basis of the analysis stated above, we constructed a robust and effective necroptosis score to predict the response to clinical immunotherapy or patient survival. Patients with a low necroptosis score or high necroptosis score showed significant differences with regard to clinicopathological characteristics, prognosis, the TME, and immune checkpoints. Our findings suggest that NRGs might serve as a clinical predictive marker for evaluating the outcome and immunotherapy response of people suffering from AML.

The TME is crucial for understanding how cancer cells grow and progress, and has a vital role in tumor biology [39]. The TME comprises tumor-infiltrating immune cells (TIICs), fibroblasts, blood vessels, and the extracellular matrix [40]. Necroptosis induction is involved in the TME, and immunosuppression of the TME reduces the resistance of tumor cells to antitumor therapies [39]. Necroptosis-induced CXCL1 expression influences the immunosuppressive TME associated with intact RIP1/RIP3 signaling [38]. The association between the number of immune cells infiltrating and the clinical prognosis and treatment responsiveness has attracted attention recently. We showed that the relative abundance of 23 TIICs differed significantly except for CD56 bright natural killer cells between the three necroptosis clusters. Moreover, most immune cells were positively related to the necroptosis score, which included activated dendritic cells, gamma delta T cells, macrophages, mast cells, and natural killer T cells. Gamma delta T cells has been reported to participate in regulating graft-*versus*-host disease and the graft-*versus*-leukemia effect [41, 42]. In addition, RIPK1 expression has been found to be upregulated in TAMs [43]. Also, inhibition of RIPK1-reprogrammed TAMs towards an immunogenic phenotype can elicit activation of cytotoxic T cells and differentiation of T-helper cells [43].

ICB therapy has shown promising clinical benefit in cancer (especially in solid tumors). Recently, immunotherapeutic drugs have been reported to lengthen survival in AML [44, 45]. Compared with their application in AML, immune-checkpoint inhibitors have provided more significant benefit in treatment of solid tumors such as melanoma and non-small-cell lung cancer [46, 47]. We wanted to identify novel molecular markers that could be used to screen AML patients and predict the response to immunotherapy precisely. We found higher expression of PD-1/PD-L1 and CD86 transcription in the group with a high necroptosis score with a poor prognosis. These data supported the potential predictive value of the necroptosis score on immunotherapy benefits. CTLA-4/CD80 and CD86 or PD-1/PD-L1 and PD-L2 participate in checkpoint control of T-cell effector functions, which can regulate T-cell activation [44]. Patients with malignant melanoma have been shown to achieve prolonged remission with the anti-CTLA-4 antibodies ipilimumab or tremelimumab [48]. We concluded that patients with a high necroptosis score, who had high expression of PD-1/PD-L1 and CD86, might respond to ICB. In addition, the necroptosis score was negatively associated with the TIDE score, MDSCs, and the M2 subtype of TAMs. The necroptosis score was positively related to IFNG, merck18, and T-cell dysfunction-score signatures. Patients with a high TIDE score are more likely to reduce the response to ICB treatment [49]. The TIDE score can help to identify patients that may be more likely to benefit from ICB [50]. The M2 subtype of TAMs contributes to immune suppression in the TME [51]. The necroptosis score was appreciably higher in responders than in non-responders. Patients with a high necroptosis score tended to benefit from ICB treatment. This finding might offer valuable insights into immunotherapy for AML patients.

While several other authors have also attempted to develop NRGs models for AML, we took a different approach to construct and validate a necroptosis score [52, 53]. There are also some limitations to this study. The prognostic model constructed for AML in this study needs to be further verified by large-sample clinical studies. At the same time, although our bioinformatics analyses provided some immunological insights of NRGs in AML and highlighted their potential role as predictive biomarkers for immunotherapy, further investigations including prospective clinical assessment are required.

Therefore, we analyzed the NRG signature among 151 AML samples. We also evaluated the association of the NRG signature with the prognosis, clinicopathological features, and TME cell-infiltrating characteristics. Evaluating the NRG patterns of an individual tumor might provide important insights into “personalized” immunotherapy strategies for patients with AML.

## Supplementary Materials

Supplementary Figures

Supplementary Tables 3-4, 6 and 13

Supplementary Tables 1-2, 5, 7-12 and 14
