# Supplementary material for: Identification and validation of necroptosis-related gene signatures to predict clinical outcomes and therapeutic responses in acute myeloid leukemia
Source: Aging (Albany NY). 2023 Nov 21;15(24):14677–702. doi: 10.18632/aging.205231 (PMC10781507; doi:10.18632/aging.205231)
Supplement: Supplementary Figures [file aging-15-205231-s001.pdf]

SUPPLEMENTARY FIGURES

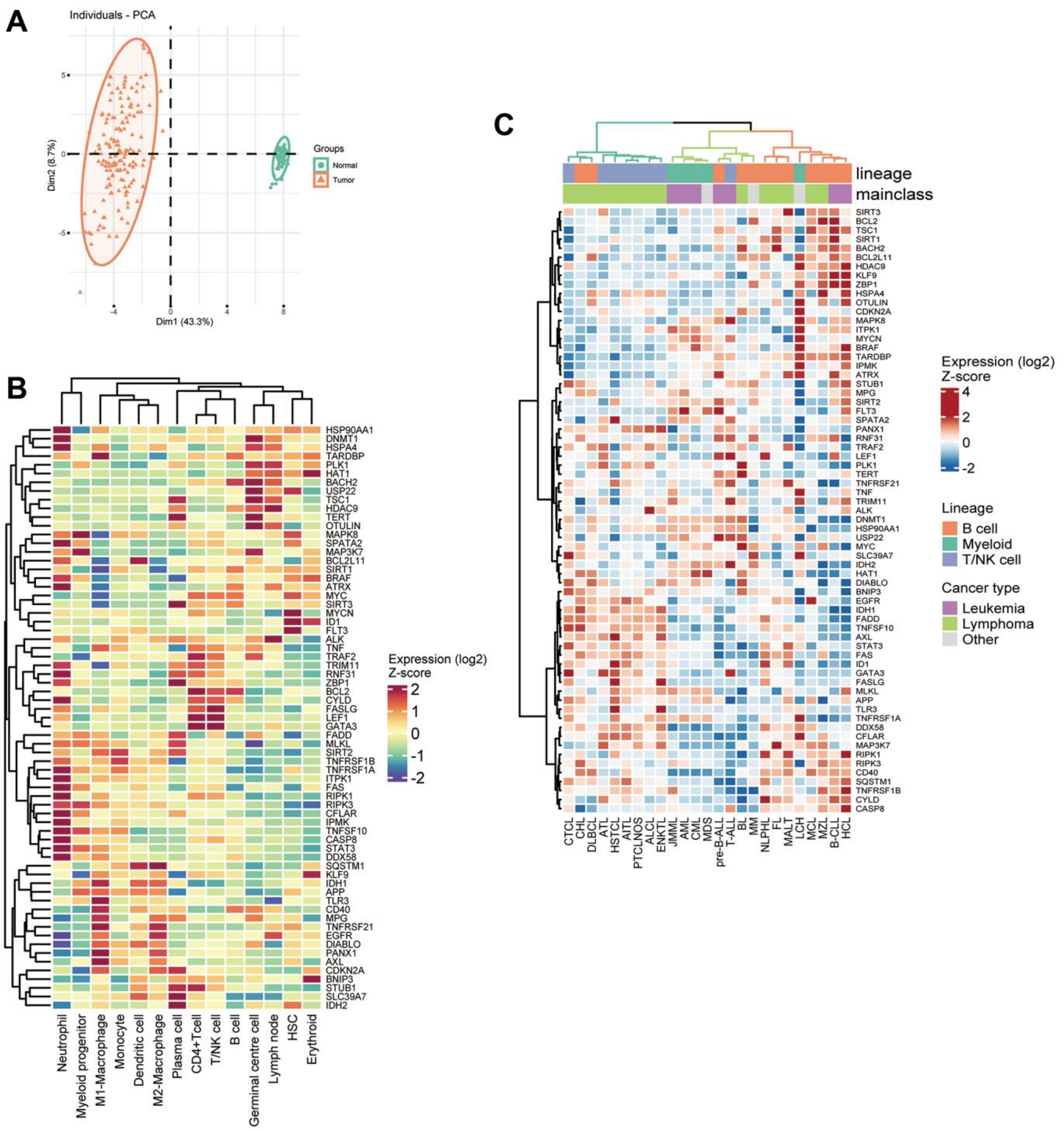

Supplementary Figure 1. Characterization of necroptosis-related genes. (**A**) Principal component analysis of 67 necroptosis-related genes based on paired tumor samples and normal samples. (**B**, **C**) Heatmap of expression of 67 necroptosis-related genes in different types of immune cells (**B**) and hematological malignancies (**C**).

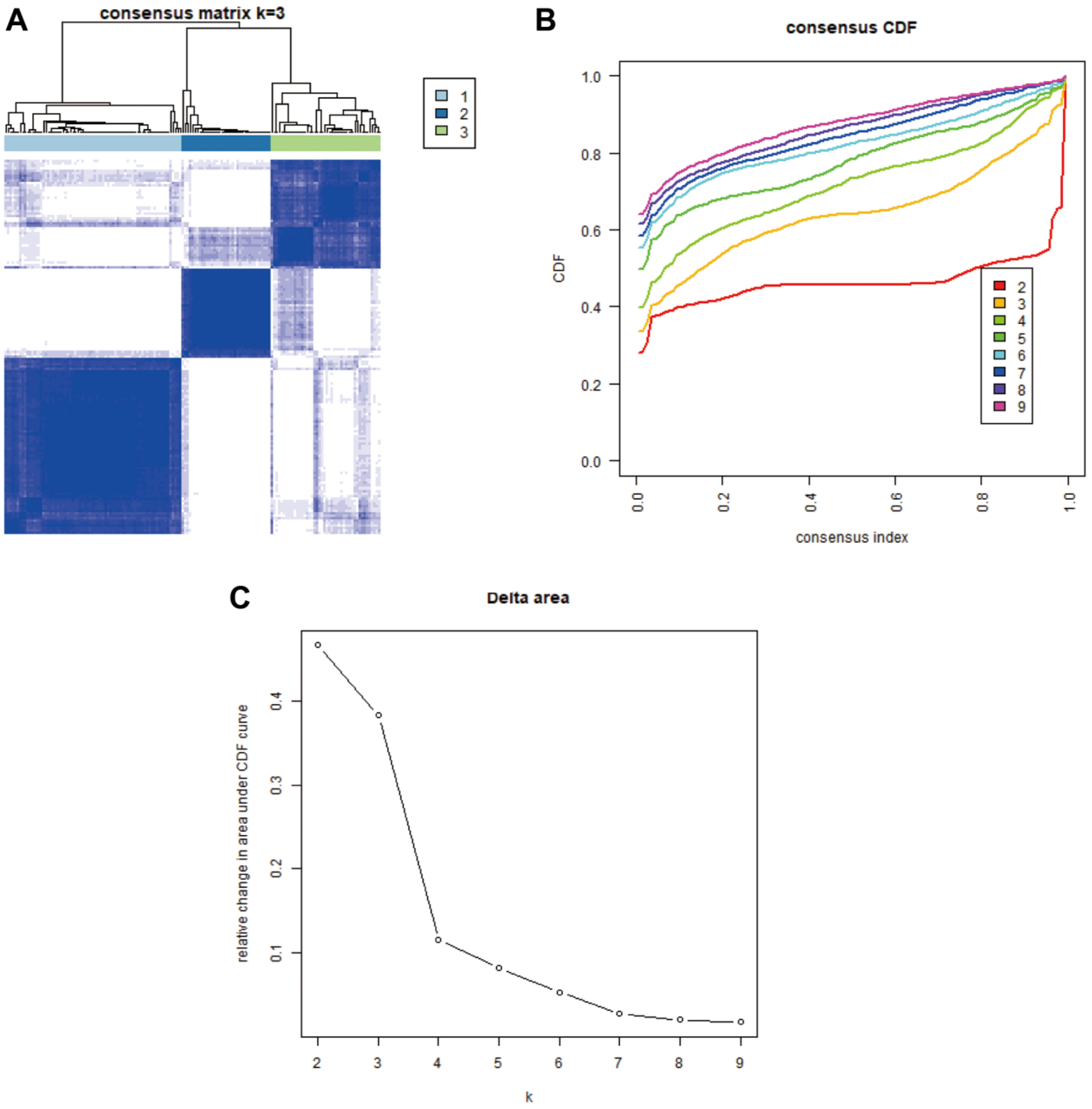

Supplementary Figure 2. Unsupervised clustering analyses of a necroptosis signature. (**A**) Concordance matrix of subtypes. (**B**, **C**) Cumulative distribution function presented as the relative change in area when k = 2–9.

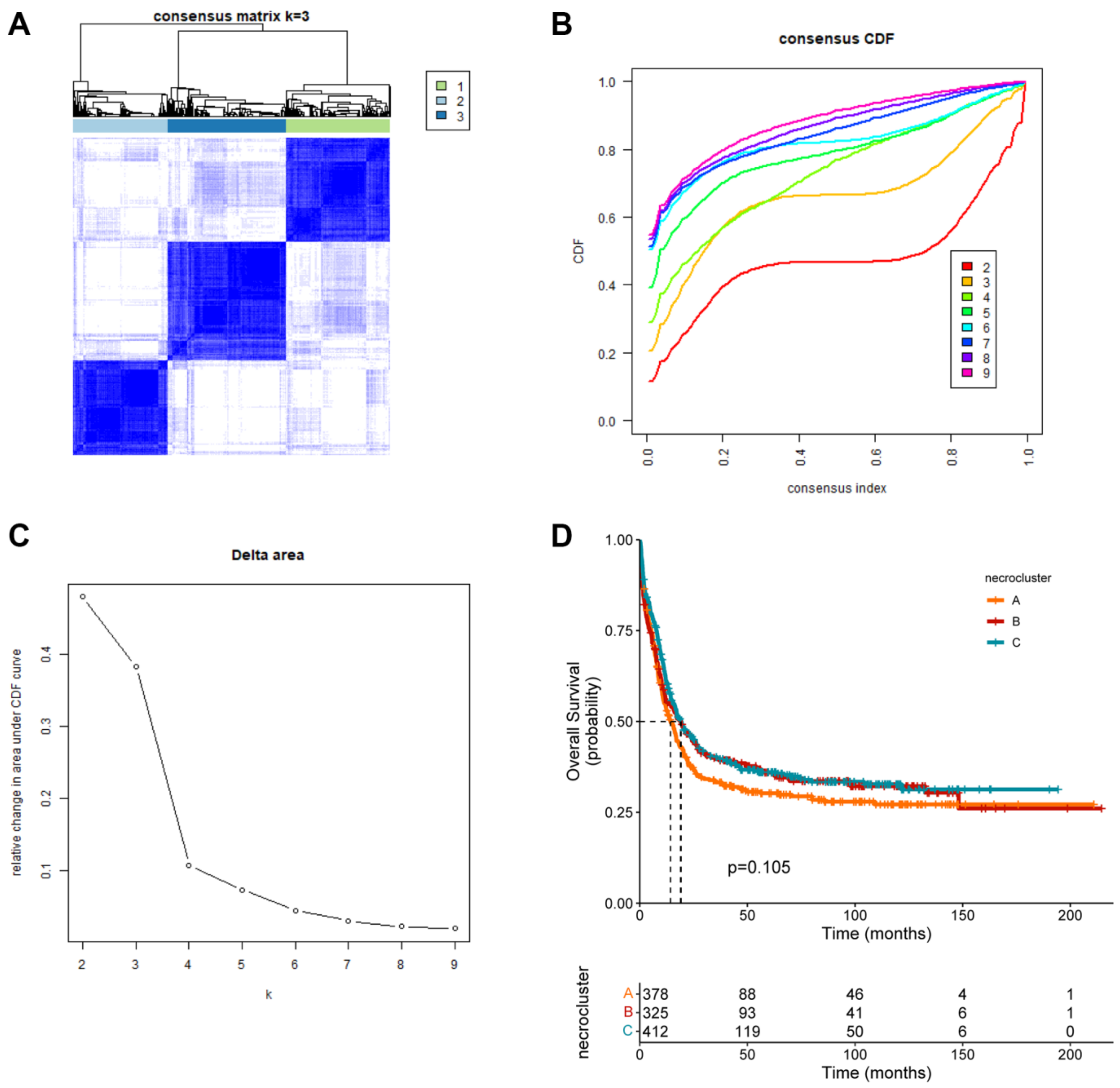

Supplementary Figure 3. Consensus clustering analysis in combined AML cohorts encompassing six datasets (1115 patients). (**A**) The concordance matrix of the subtypes was calculated. (**B**, **C**) The cumulative distribution function and relative change in area under the cumulative distribution function curve for k = 2–9. (**D**) Kaplan–Meier curves showing the overall survival for three clusters (*P* = 0.105, log-rank test).

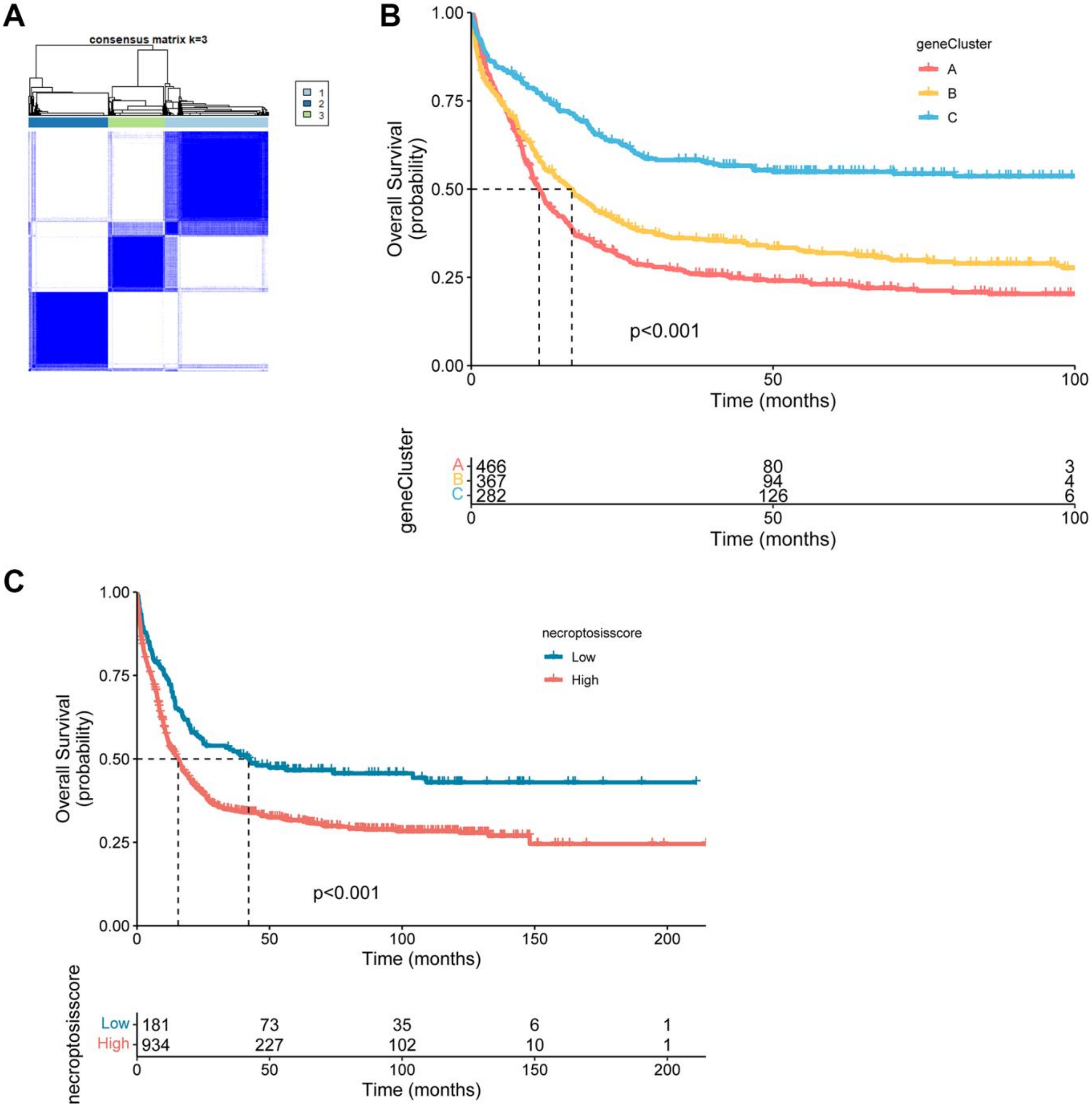

Supplementary Figure 4. Prognosis analysis of three gene clusters and the necroptosis score in combined AML cohorts encompassing six datasets (1115 patients). (**A**) Consensus matrix heatmap defining three gene clusters (k = 3). (**B**) Kaplan-Meier overall survival curves of three gene clusters (P < 0.001, log-rank test). (**C**) Kaplan–Meier survival analysis of high versus low necroptosis score groups (P < 0.001, log-rank test).
