# Supplementary material for: Identification and validation of necroptosis-related gene signatures to predict clinical outcomes and therapeutic responses in acute myeloid leukemia
Source: Aging (Albany NY). 2023 Nov 21;15(24):14677–702. doi: 10.18632/aging.205231 (PMC10781507; doi:10.18632/aging.205231)
Supplement: Supplementary Tables 3-4, 6 and 13 [file aging-15-205231-s002.pdf]

SUPPLEMENTARY TABLES

Supplementary Table 3. The locations of 67 necroptosis-related genes on their respective chromosomes.

Chromosome	chromStart	chromEnd	Gene
chr1	11012344	11026420	TARDBP
chr1	12167003	12209228	TNFRSF1B
chr1	172659018	172666874	FASLG
chr1	228393673	228406840	TRIM11
chr2	15940564	15947007	MYCN
chr2	29192774	29921566	ALK
chr2	111119378	111168447	BCL2L11
chr2	171922448	171983682	HAT1
chr2	201116104	201176687	CFLAR
chr2	201233443	201287711	CASP8
chr2	208236227	208266074	IDH1
chr3	172505508	172523507	TNFSF10
chr4	108047545	108168956	LEF1
chr4	186069152	186088069	TLR3
chr5	1253147	1295069	TERT
chr5	14664664	14699711	OTULIN
chr5	133051962	133106449	HSPA4
chr5	179806398	179838078	SQSTM1
chr6	3063991	3115187	RIPK1
chr6	31575567	31578336	TNF
chr6	33200445	33204439	SLC39A7
chr6	47231532	47309905	TNFRSF21
chr6	89926529	90296908	BACH2
chr6	90513573	90587045	MAP3K7
chr7	18086949	19002416	HDAC9
chr7	55019021	55256620	EGFR
chr7	140719327	140924764	BRAF
chrX	77504878	77786269	ATRX
chr8	127735434	127741434	MYC
chr9	21967753	21995301	CDKN2A
chr9	32455705	32526324	DDX58
chr9	70384597	70414624	KLF9
chr9	132891348	132944633	TSC1
chr9	136881912	136926607	TRAF2
chr11	215458	236931	SIRT3
chr11	70203163	70207390	FADD
chr11	94128928	94181972	PANX1
chr10	8053604	8075198	GATA3
chr10	48306639	48439360	MAPK8
chr10	58191517	58267934	IPMK
chr10	67884669	67918390	SIRT1

chr10	88990531	89015785	FAS
chr10	131966455	131982013	BNIP3
chr12	6328757	6342114	TNFRSF1A
chr12	122207662	122227534	DIABLO
chr13	28003274	28100592	FLT3
chr14	24146683	24160661	RNF31
chr14	24336021	24340045	RIPK3
chr14	92936914	93116320	ITPK1
chr14	102080738	102139699	HSP90AA1
chr15	90083045	90102504	IDH2
chr16	77007	85853	MPG
chr16	680224	682870	STUB1
chr16	23677656	23690367	PLK1
chr16	50742050	50801935	CYLD
chr16	74671855	74700960	MLKL
chr17	20999593	21043760	USP22
chr17	42313324	42388568	STAT3
chr18	63123346	63320128	BCL2
chr20	31605283	31606515	ID1
chr20	46118272	46129863	CD40
chr20	49903391	49915508	SPATA2
chr20	57603846	57620576	ZBP1
chr19	10133345	10231286	DNMT1
chr19	38878555	38899862	SIRT2
chr19	41219203	41261766	AXL
chr21	25880550	26171128	APP

Supplementary Table 4.The CNV alterations of 67 necroptosis-related genes on their respective chromosomes.

chromosome	start	stop	seg.mean
chr1	11012344	11026420	1
chr1	12167003	12209228	1
chr1	172659018	172666874	0
chr1	228393673	228406840	1
chr2	15940564	15947007	0
chr2	29192774	29921566	0
chr2	111119378	111168447	0
chr2	171922448	171983682	0
chr2	201116104	201176687	−1
chr2	201233443	201287711	−1
chr2	208236227	208266074	0
chr3	172505508	172523507	0
chr4	108047545	108168956	0
chr4	186069152	186088069	0
chr5	1253147	1295069	0

chr5	14664664	14699711	0
chr5	133051962	133106449	−1
chr5	179806398	179838078	−1
chr6	3063991	3115187	0
chr6	31575567	31578336	0
chr6	33200445	33204439	0
chr6	47231532	47309905	0
chr6	89926529	90296908	0
chr6	90513573	90587045	0
chr7	18086949	19002416	−1
chr7	55019021	55256620	0
chr7	140719327	140924764	−1
chrX	77504878	77786269	0
chr8	127735434	127741434	1
chr9	21967753	21995301	−1
chr9	32455705	32526324	0
chr9	70384597	70414624	−1
chr9	132891348	132944633	1
chr9	136881912	136926607	1
chr11	215458	236931	0
chr11	70203163	70207390	1
chr11	94128928	94181972	1
chr10	8053604	8075198	0
chr10	48306639	48439360	0
chr10	58191517	58267934	0
chr10	67884669	67918390	0
chr10	88990531	89015785	0
chr10	131966455	131982013	0
chr12	6328757	6342114	0
chr12	122207662	122227534	0
chr13	28003274	28100592	−1
chr14	24146683	24160661	−1
chr14	24336021	24340045	−1
chr14	92936914	93116320	0
chr14	102080738	102139699	0
chr15	90083045	90102504	0
chr16	77007	85853	0
chr16	680224	682870	0
chr16	23677656	23690367	0
chr16	50742050	50801935	1
chr16	74671855	74700960	−1
chr17	20999593	21043760	0
chr17	42313324	42388568	−1
chr18	63123346	63320128	0
chr20	31605283	31606515	1

chr20	46118272	46129863	−1
chr20	49903391	49915508	−1
chr20	57603846	57620576	1
chr19	10133345	10231286	1
chr19	38878555	38899862	1
chr19	41219203	41261766	1
chr21	25880550	26171128	−1

Supplementary Table 6. The results of univariate Cox regression analysis of 67 necroptosis-related genes in AML.

ID	HR	HR.95L	HR.95H	*P*-value	km
SIRT1	0.653185005	0.378242712	1.127981155	0.126532966	0.0141689
SIRT2	2.184984848	1.310189482	3.643868962	0.00274144	0.000156203
IPMK	0.832128264	0.576823465	1.200432177	0.325662055	0.114827499
FLT3	1.145843585	0.941964664	1.39385008	0.173235112	0.005395313
DDX58	1.078687703	0.714994691	1.627378742	0.718089398	0.089256771
AXL	0.893148058	0.616525233	1.293886138	0.550144066	0.003022712
HAT1	1.879445439	1.059558298	3.333761968	0.030942926	0.002192668
TRIM11	1.692538231	0.908277659	3.1539757	0.097514088	0.01034464
MYC	0.991110343	0.817531286	1.201543879	0.927573176	0.164023709
PLK1	0.978689975	0.754347237	1.269752204	0.871185873	0.02396733
MPG	1.377101107	0.988070387	1.919304011	0.058877071	0.000623289
TNFRSF1B	1.163894293	1.030802172	1.314170617	0.014301022	0.002521854
CASP8	1.300459795	0.701888456	2.409493508	0.403741253	0.148505378
RNF31	1.311566439	0.7462553	2.305117998	0.345845229	0.142074969
TSC1	0.865754449	0.518796244	1.444749792	0.581131898	0.003174917
PANX1	1.781511256	1.21093007	2.620946026	0.003372425	0.003850982
TLR3	0.768912602	0.442353635	1.336547374	0.351558403	0.103295508
DIABLO	0.448302915	0.191224639	1.050991677	0.064956574	0.00054681
EGFR	0.162124498	0.009434344	2.786028751	0.209898964	0.002225816
MYCN	0.900484343	0.792057853	1.023753566	0.109303252	0.03588317
SLC39A7	0.828131159	0.477791165	1.435357676	0.501561935	0.017329371
FAS	0.885475167	0.594179178	1.319578841	0.55013449	0.029230075
SQSTM1	1.135111031	0.772580721	1.66775719	0.518550154	0.015239662
BACH2	1.133161573	0.874854048	1.467736424	0.343599343	0.040783794
ATRX	0.916901721	0.617217044	1.362095836	0.66746871	0.198735659
HDAC9	1.155183906	0.95236621	1.40119404	0.14305802	0.002137129
ZBP1	1.68624203	1.124616735	2.52833885	0.011463859	0.001224679
MAPK8	0.697286568	0.417564651	1.164391087	0.16814127	0.223565352
BNIP3	0.577742784	0.381540903	0.874838641	0.009552699	0.003061902
RIPK1	1.045134946	0.493019116	2.215547064	0.908319629	0.044349595
ALK	2.600097719	0.8630238	7.833512993	0.08947663	0.001249055
BCL2L11	1.468472638	1.126975312	1.913450868	0.004439423	0.002552374
BRAF	0.480067011	0.27963122	0.824172405	0.007785274	0.001083898

HSPA4	1.301501392	0.809385541	2.092829423	0.276884707	0.006134598
TERT	1.32265337	0.953263779	1.83518138	0.094224722	0.001181906
MLKL	1.043130146	0.728780155	1.49307098	0.817482411	0.149133862
BCL2	0.823515447	0.640900027	1.058164554	0.129018388	0.007601644
ITPK1	1.897746809	1.33293067	2.701898179	0.000379003	1.31422E-05
CYLD	1.445531804	0.814540202	2.565327272	0.208012226	0.015127248
DNMT1	1.519224994	0.89849651	2.568785251	0.118627464	0.001731079
FADD	2.752830445	1.553552027	4.877902591	0.000521882	1.1015E-07
TNF	0.878746969	0.738113019	1.046176149	0.146321965	0.009964063
KLF9	1.296480875	1.068129904	1.573650033	0.008621241	0.001611377
IDH2	1.259520778	0.806763049	1.966367439	0.310013134	0.099546954
TRAF2	1.056712493	0.638818732	1.74797832	0.829909929	0.036640337
HSP90AA1	1.316781385	0.903432252	1.919250958	0.15224634	0.007182229
RIPK3	1.420721389	1.033504631	1.953014245	0.030545089	0.001200498
FASLG	1.041890084	0.639615345	1.697168392	0.869069192	0.028144583
CD40	1.206387454	0.932591082	1.560566809	0.153119006	0.000667904
SPATA2	0.746521175	0.406950179	1.369440027	0.344998987	0.055968205
ID1	1.200475632	1.006408247	1.431965354	0.042258402	0.000194623
LEF1	0.917595921	0.749311916	1.12367394	0.405440668	0.012767755
GATA3	1.015865859	0.786799863	1.31162128	0.903895127	0.06558502
CDKN2A	1.081608681	0.843680642	1.386635276	0.535970717	0.002878496
STAT3	0.683169486	0.389810292	1.197301752	0.183207074	0.030650878
TNFRSF21	1.106554528	0.929281323	1.317645037	0.25569587	0.076153834
IDH1	1.42470344	1.011777604	2.006152228	0.0426608	0.000541714
CFLAR	0.941588305	0.597826846	1.48301894	0.795109628	0.217209513
SIRT3	0.839786189	0.481646278	1.464229821	0.538170095	0.048744659
MAP3K7	1.055232867	0.516374652	2.156411823	0.882787486	0.084903579
TARDBP	1.462416141	0.705296405	3.032286788	0.306980494	0.033807403
APP	0.912122011	0.828108786	1.004658539	0.062084379	0.000465397
TNFSF10	1.015918702	0.860748485	1.199062012	0.851850785	0.162765083
OTULIN	0.765242099	0.427404261	1.370120806	0.36793924	0.051735805
TNFRSF1A	0.811636879	0.482530203	1.365208682	0.431505516	0.07905431
USP22	0.997466291	0.578531952	1.719765	0.992716961	0.241687143
STUB1	1.516106553	0.973929262	2.36010886	0.065332889	0.000233725

Supplementary Table 13. The univariate analyses of necroptosisscore in the TCGA.

Beta	HR (95% CI for HR)	wald.test	*P*-value	variables
0.03893	1.04 (1.025–1.055)	26.96	2.074e-07	AGE
1.183	3.264 (2.175–4.898)	32.65	1.104e-08	AGEcat
0.005017	1.005 (1.001–1.009)	5.42	0.01995	WBC
0.2223	1.249 (0.8345–1.869)	1.17	0.28	WBCcat
0.146	1.157 (0.667–2.008)	0.27	0.6034	FLT3_n
−0.02775	0.9726 (0.4897–1.932)	0.01	0.9368	CEBPA
0.1453	1.156 (0.7373–1.813)	0.4	0.5269	NPM_n

1.63	5.105 (2.642–9.861)	23.55	1.219e-06	TP53
−0.2314	0.7934 (0.3846–1.637)	0.39	0.531	IDH1_n
0.01886	1.019 (0.5422–1.915)	0	0.9533	IDH2_n
0.6019	1.826 (1.159–2.876)	6.74	0.009435	DNMT3A
0.7677	2.155 (1.164–3.988)	5.98	0.01451	RUNX1
0.6329	1.883 (1.39–2.551)	16.71	4.344e-05	RISK
2.038	7.673 (2.424–24.29)	12.01	0.0005283	necroptosisscore_group
0.02592	1.026 (1.012–1.041)	12.74	0.000357	necroptosisscore
